# Use of quarry waste basalt rock powder as a soil remineralizer to grow soybean and maize

**DOI:** 10.1016/j.heliyon.2023.e14050

**Published:** 2023-02-26

**Authors:** Augusto Vaghetti Luchese, Ivone Janete Gutz de Castro Leite, Ana Paula da Silva Giaretta, Mylena Linhares Alves, Laércio Augusto Pivetta, Robson Fernando Missio

**Affiliations:** a Department of Agronomic Sciences, Federal University of Paraná (UFPR), Setor Palotina, Rua Pioneiro, 2153, Jd Dallas, Palotina, Paraná, Brazil; b Graduate Program in Biotechnology, Federal University of Paraná (UFPR), Setor Palotina, Rua Pioneiro, 2153, Jd Dallas, Palotina, Paraná, Brazil

**Keywords:** Basalt rock powder, Soybean, Maize, Fertilizer, Stone meal

## Abstract

Production costs in Brazilian agriculture have increased with the rising prices of imported soluble fertilizers. To circumvent this import dependence, low-cost indigenous nutrient sources have been tested, including basalt rock powder (BRP). In this study, we assessed BRP and limestone effects on soil fertility, and soybean and maize dry mass (DM) accumulation. Four greenhouse pots experiments were arranged in a 2 × 4 factorial design with two soils (Clay and Sandy Clay Loam) and four doses of each material (0, 33, 66, and 99 Mg ha^−1^ BRP and 0, 1, 2, and 4 Mg ha^−1^ limestone), evaluated in two species (soybean and maize). At the end of the experiments, DM, shoot P and K concentrations, and soil pH and P, K, Ca, and Mg concentrations were assessed as a function of BRP and limestone application. Applying BRP increased DM production and improved soil fertility parameters such as pH, and Ca and P concentrations, with leaf P content also increasing. Meanwhile, limestone only triggered significant changes in pH and soil Ca content.

## 1 Introduction

More than 80% of the fertilizers used in Brazilian agriculture are imported [1,2], a fact that reveals an inconvenient overdependence of Brazilian agriculture on fertilizer imports and the resulting vulnerability to foreign fertilizer-market trends. Indeed, such overdependence on fertilizer imports is a serious cause for concern, as agribusiness is the largest Brazilian production chain, and being alarmingly intertwined with basic imported materials makes it highly susceptible to exchange rate variations. Further, Brazil is the fourth largest consumer of fertilizers worldwide, accounting for 8% of global consumption, only behind China, India, and the United States. Particularly, soybean, maize, and sugarcane cropping account for more than 73% of the total fertilizer consumption in the country [3]. Therefore, the high demand for fertilizers in Brazil has stimulated consideration of alternative materials to use as soil media and plant nutrient sources. A key example is the soil remineralizer basalt rock powder (BRP), found in quarry waste derived from the processing of specific basaltic rocks, which has been tested as a fertilizer for several crops [[4], [5], [6], [7]].

According to Decree 8384/2014 of the Ministry of Agriculture, Livestock and Food Supply (Ministério da Agricultura, Pecuária e Abastecimento – MAPA), BRP is defined as a mineral material that has only undergone particle size reduction and classification through mechanical processes and, when applied to the soil, alters its fertility indices by providing plants with macronutrients and micronutrients, and by improving soil physical and physicochemical properties or biological activity. Through this decree, MAPA regulates the use of these materials in Brazilian agriculture.

Soil remineralizer BRP, commonly known as rock powder, rejuvenates the soil because rock grinding releases macro and micronutrients [5,8]. Studies have shown that applying BRP significantly improves the chemical properties of the soil, particularly calcium, magnesium, phosphorus, and potassium concentrations [5,[9], [10], [11]].

Soil remineralizers help to neutralize soil acidity [4,6,12] either through the reaction of calcium and magnesium oxides present in rock powders, thereby releasing OH^−^, or through H^+^ consumption during silicate mineral alteration, as that occurring in microcline reactions, as explained by Refs. [6,13]. Although BRP acts differently from limestone in the pH correction, it can impair the plant growth due to reduced availability of nutrients, especially cationic micronutrients, whose availability tends to decrease with increasing soil pH [9].

Natural transformations of primary minerals of these rocks, such as biotite, during their weathering processes, also form 2:1 secondary minerals, such as vermiculite [14], promoting cation exchange capacity (CEC) gains in soils treated with these rock powders [15,16]. Dozens of Brazilian rocks can be used for this purpose, such as basalt, diabase, phonolite, phosphorite, breccia, and biotite schist [5,13,15].

**Fig. 1:**
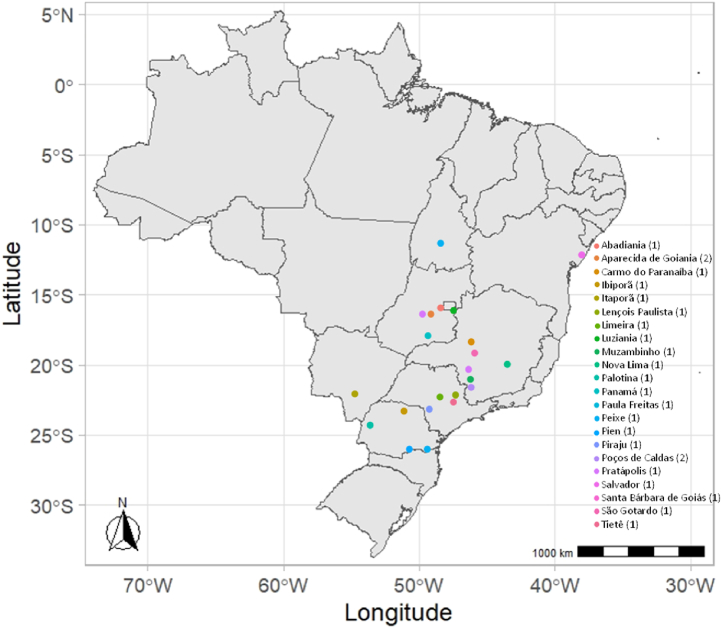
Map of Brazil indicating the location of mining companies that produce BRP and other MAPA-certified products from silicate rocks. The numbers in parentheses represent the number of mining companies producing BRP.

Although studies have been conducted on various materials, the number of publications on remineralization remains low compared with research on soluble fertilizers. In addition, significant differences occur both between and within rock types, depending on their place of origin same rocks could present significant chemistry and mineralogy differences, thereby generating the demand for specific studies on each material derived from mining. In Brazil, various mining companies use rocks as the raw material of numerous products, generating large amounts of waste, such as mining tailings (Fig. 1). Therefore, using BRP as an alternative fertilization source has considerable environmental importance for both low-cost fertilizer production and sustainable waste (which is generated in large quantities) disposal.

Based on the above, the present study aimed to assess the effect of applying BRP on the chemical properties of the soil and on the initial growth and nutritional status of soybean and maize plants.

## 2 Materials and methods

The experiments were conducted in a greenhouse with temperature control (adjusted to 28 °C) and plastic cover with 20% of light reduction, at the Federal University of Paraná (Universidade Federal do Paraná – UFPR), Palotina Sector, in Palotina, Paraná. The study comprised four experiments arranged in a 2 × 4 factorial design with two soils (Clay and Sandy Clay Loam) and four doses of each material (0, 33, 66, and 99 Mg ha^−1^ BRP and 0, 1, 2, and 4 Mg ha^−1^ limestone, evaluated independently) evaluated in two species (soybean and maize), with four replications.

Due to the fact that the pH influences the dynamics of several nutrients in soil, it is important to compare BRP with another soil acidity corrective, in this case limestone, to distinguish whether the effect on crop growth and on the availability of P and K is derived from the increase in soil pH or from the release of constituent nutrients from the BRP minerals. Limestone has the purpose of reducing soil acidity and does not contain significant amounts of micronutrients and macronutrients, such as K and P, in its constitution.

The material called RM Fortaleza, provided by the company Goyaz Britas, was used for the re-mineralizing treatment. Chemical composition and particle size are summarized in Table 1, Table 2. The limestone consisted of calcitic limestone with 45% CaO and 4% MgO, and 72.5% effective calcium carbonate equivalent (ECCE).

**Table 1:** Chemical composition of the BRP used in the experiment, as per X-ray fluorescence spectrometry (XRF).

SiO_2_	Al_2_O_3_	Fe_2_O_3_	CaO	MgO	K_2_O	Na_2_O	TiO_2_	MnO	P_2_O_5_	LOI	Sum
64.05	9.66	9.03	4.89	2.70	3.32	1.70	2.11	0.12	0.30	2.52	100.4

SiO_2_: silicon dioxide; Al_2_O_3_: aluminum oxide; Fe_2_O_3_: iron oxide; CaO: calcium oxide; MgO: magnesium oxide; K_2_O: potassium oxide; Na_2_O: sodium oxide; TiO_2_: titanium dioxide; MnO: manganese oxide; P_2_O_5_: phosphorus pentoxide; LOI: loss on ignition.

**Table 2:** Particle size as determined by screening the Basalt Rock Powder (BRP) and Limestone used.

Sieve mesh (mm)	4.75	2	1	0.3	0.075	<0.075
**BRP Particle retention (%)**	0	0.35	7.93	21.15	31.97	38.6
**Limestone Particle Retention (%)**	0	0	0	0	100	–

The composition of BRP as per X-ray diffraction analysis with monochromatic radiation from a copper anode tube and Rietveld refinement demonstrated the presence of the following crystalline mineral phases: labradorite (26.97%), augite (25.89%), andesite (8.2%), microcline (6.12%), chlorite (4.67%), ilmenite (3.89%), analcime (2.03%), quartz (2.02%), magnetite (1.95%), calcite (1.59%), apatite (0.52%), and celadonite (0.52%). Additionally, low-crystallinity materials were also present (14.20%).

The study was conducted in subsurface soil samples collected at depths >0.20 m (aiming soils with low fertility) on farms in the municipality of Guaíra-PR (sandy clay loam) and Cascavel-PR (clay) (Table 3). After collection, soils were air-dried, passed through a 4 mm sieve, and placed into 5 L plastic pots. Treatment quantities were calculated for a volume ratio of 2,000,000 L soil ha^−1^.

**Table 3:** Chemical and particle size analysis of the clay and sandy clay loam (SCL) experimental soils.

Texture	pH^b^	Al^3+^^c^^,^^d^	H + Al^e^^,^^f^	Ca^2+^^d^	Mg^2+^^d^	K + g	P^7^	O.C^h^	Sand	Silt	Clay
		--------- cmol_c_ dm^−3^ ----------					mg dm^−3^	g dm^−3^	----- g kg^−1^ -----		
Clay	4.5	0	5.74	2.9	1.1	0.16	1.30	22.4	274	113	612
SCL^a^	5.2	0	2.03	3.4	1.5	0.45	14.6	11.3	693	13	294

a Sandy Clay Loam.

b pH in CaCl_2_ 0,02 M.

c Exchangeable potential acidity.

d Extraction by KCl 1 M.

e Total potential acidity.

f Determined by pH SMP buffer.

g Extraction by Mehlich I.

h Organic Carbon, determined by Walckley Black.

To promote the treatment reactions in the soil, pots were kept moist for 30 days. Subsequently, five soybean (Monsoy 6210 cultivar) or maize (Morgan 618) seeds were sown in the respective pots. At 20 days after sowing, thinning was performed, leaving only two plants per pot. The plants were manually irrigated every day throughout the study period. In the maize plants, nitrogen fertilization was performed with urea at a dose equivalent to 100 kg of N ha^−1^, at 34 days after sowing. Approximately 150 mL of soil was collected from the pots after homogenization. In addition to soil sampling for chemical analysis, entire plants were collected 47 days after sowing by cutting the shoots close to soil surface (soybean at R2, and maize at V10). The collected materials were oven-dried at 50 °C under forced-air circulation for approximately 48 h, followed by weighing (only plant tissue) and milling for tissue nutrient analysis. Shoot samples were digested with a nitro-perchloric solution prior to P and K determination by colorimetry and flame photometry, respectively [17]. Soil samples were analyzed to measure the pH in CaCl_2_ by potentiometry, P by spectrophotometry, and K by photometry, using Mehlich I, and Ca and Mg (volumetry) using 1 M KCl, after [18].

### 2.1 Statistical analysis

After all assessments were concluded, the data were tabulated and tested for homogeneity of variance and normal distribution (Kolmogorov-Smirnov and Shapiro-Wilk). Subsequently, data were submitted to analysis of variance (ANOVA) by the F test and, in cases of significance for the doses (quantitative factor), regression analysis was performed to test the best fit model of the data, as recommended by Ref. [19]. All analyses were performed using the Sisvar software [19].

## 3 Results and discussion

### 3.1 Effects on dry matter (DM) and pH

**Fig. 2:**
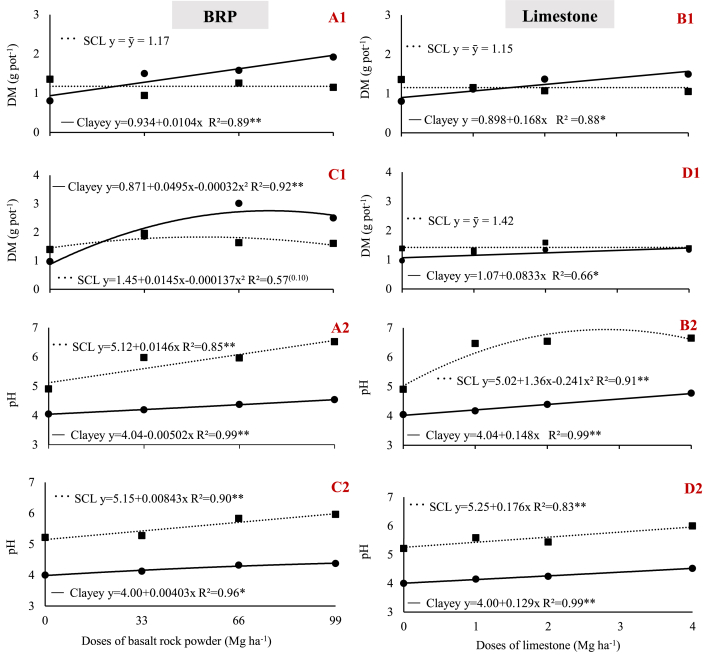
Variation of shoot dry matter (DM) mass (1) and soil pH (2) as a function of BRP and Limestone dose, with: A) BRP with soybean; B) Limestone with soybean; C) BRP with maize; D) Limestone with maize; in sandy clay loam (SCL) and clay (Clayey) soils. ** and * indicate significant differences at 1% and 5% probability, respectively.

In the clay soil, shoot dry matter (DM) of both plant species tested increased with BRP dose, peaking at 1.92 g in soybean plants supplied with 99 Mg ha^−1^ and at 2.6 g in maize plants supplied with 77 Mg ha^−1^ (Fig. 2 A1, C1). Initially, the clay was less fertile than the sandy-clay loam soil (Table 3), in which case, soybean DM remained unaffected by BRP application, whereas maize DM tended to increase as BRP dose increased up to 53 Mg ha^−1^ (Fig. 2 A1 and C1). Corroborating the results, soybean DM was shown to increase in a clay soil when using biotite schist and alkaline and ultramafic alkaline breccia [20].

Although limestone also increased DM in the experimental clay soil, the increases observed were smaller than those observed upon BRP application, with 1.49 and 1.33 g DM in soybean and maize plants, respectively. No significant increase in DM was observed in any plant grown in the sandy-clay loam (Fig. 2 B1 and C1).

The BRP, as well as limestone, are reportedly associated with increases in soil pH [5,9,15,16,20]. Consistently, in our study, both limestone and BRP significantly increased the pH of both soils. Yet, despite the similarities between the increases generated by both materials, the limestone doses were considerably lower (Fig. 2 A2, B2, C2, and D2). However, BRP tends to have a much slower reaction time than limestone, which may be an added benefit of long-term rock powders.

Soil pH correction also had a stronger effect on the sandy-clay loam because its CEC is lower than that of the clay soil, i.e., 7.38 versus 9.9 cmol_c_ dm^−3^ respectively, regardless of material (BRP or limestone), as shown by higher slopes for the regression equations (Fig. 2 A2, B2, C2, and D2). As an acidity corrector, BRP affects soils through its carbonate minerals, with calcite accounting for 1.59% of all the constituent minerals. At this concentration of calcite, 99 Mg ha^−1^ BRP corresponds to 1.57 Mg CaCO_3_, whereas the amount of limestone applied in this study was equivalent to a dose of 2.9 Mg CaCO_3_. However, the increase in pH in both crops were very similar upon application of either BRP or limestone, with 4.5 in soybean and 4.4 in maize treated with BRP and 4.8 in soybean and 4.5 in maize treated with limestone (Fig. 2 A2, B2, C2, and D2). These results show that the pH increase under BRP treatment also resulted from H+ consumption in the reaction of Ca, Mg, Na, and K silicates [6,16]. The BRP has as its main compounds calcium silicates as labradorite (26.97%). Due to the Ca shows weaker bonds with Si, compared to Mg, K, and Na, probably the labradorite will be the main responsible by H+ consumption and pH increase by BRP [21]. The pH increase may partly explain the gain in DM in soybean and maize growing in clay soil, which initially had a low pH [22] because, although the BRP materials caused similar increases in pH, DM production increased more when BRP was applied.

### 3.2 Ca^2+^ and Mg^2+^ effects on soil

**Fig. 3:**
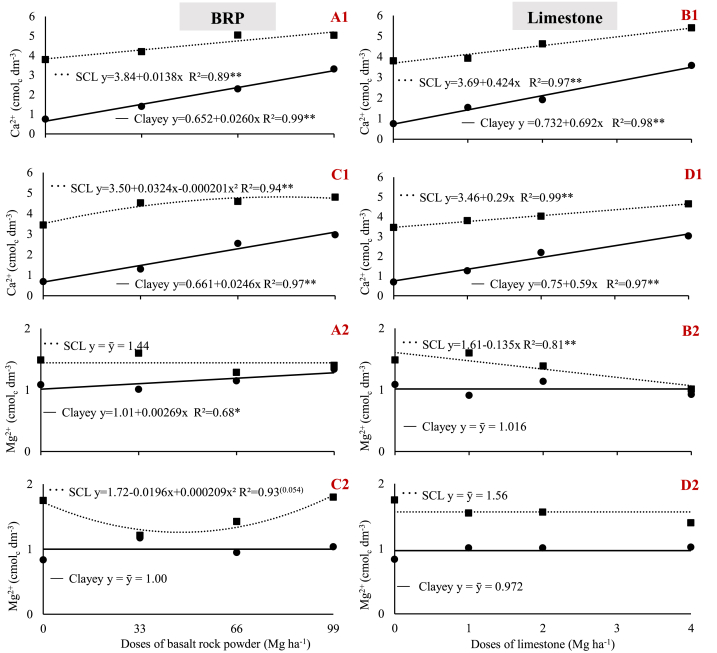
Variation of soil calcium (1) and magnesium (2) concentrations as a function of BRP and limestone dose. A) BRP with soybean; B) Limestone with soybean; C) BRP with maize; D) Limestone with maize; in a sandy-clay loam (SCL) and a clay (Clayey) soil. ** and * indicate significant differences at 1% and 5% probability, respectively.

In the clay soil, both BRP and limestone improved soil fertility by increasing the calcium concentration from low to high values (Table 3), regardless of plant species (Fig. 3 A1, B1, C1, and D1) [22]. In turn, in the sandy-clay loam, the baseline calcium concentrations were already high (Table 3). Therefore, although calcium increased on average 1.35 cmol_c_ dm^−3^, the soil calcium concentrations were still classified as ‘high’, because their classification as ‘very high’ would require an increase greater than 2 cmol_c_ dm^−3^ (Fig. 3 A1, B1, C1, and D1) [22].

Despite limestone has been applied at a final dose 24.75 times lower than BRP, it has a CaO concentration 9.2 times higher than BRP. In addition, not all Ca in BRP is present in the form of carbonate (CaCO_3_). Thus, limestone Ca predominantly derives from carbonates, which are the most soluble forms. Conversely, in BRP, which derives from basalt, calcite only partly accounts for Ca. Most of this Ca derives from other minerals, such as labradorite (26.97%), augite (25.89%), and andesine (8.20%), and the plagioclases labradorite and andesine are silicates typical of basalts [[23], [24]]. This difference in constituents ensures the increased solubility of Ca from limestone applied to the soil. The increased concentration in soil Ca released from BRP observed in this study corroborate reports on stonemeal, demonstrating that calcium is potentially released from these materials [9,12,15].

Unlike Ca, initial soil Mg concentrations were considered intermediate for the clay and high for the sandy-clay loam soil (Table 3) [22]. Such intermediate Mg concentrations, combined with the low Mg concentration of calcitic limestone (only 4% MgO), reduced the possibility of a positive response to limestone. Thus, when applying limestone, the only significant response was the decrease in Mg concentrations in the sandy clay loam cultivated with soybean (Fig. 3 B2 and D2).

The BRP-MgO mean concentration (2.7%) was not much lower than that which is characteristic of limestone (4.0%). Nonetheless, although the amount of Mg applied was higher when BRP was used (1612 kg of magnesium ha^−1^ at 99 Mg of rock dust ha^−1^) than when limestone was used (96 kg of magnesium ha^−1^ at 4 Mg of limestone ha^−1^). BRP Mg was not released due to the compounds in which it is present. Specifically, in limestone, Mg is predominantly found in the form of carbonates that are relatively more soluble than the silicate forms of BRP minerals, such as chlorite (4.67%) and augite (25.89%), and, in this study, BRP augite was predominantly Fe-Augite.

The results of BRP application to provide soils with Mg are controversial. Some authors have reported increases in soil Mg concentrations when applying rock powders [6,20], whereas others found no increase in this element [4,9]. As demonstrated in the literature, the application of rock powders may have a significant effect on soil Mg concentrations, primarily depending on the minerals present that will supply the soil with this nutrient and on the concentration of each mineral.

Elements are preferentially released from rocks in the following order: CaO > MgO » Al(OH)_3_>FeOOH > Si_2_. This factor, combined with the formation of secondary minerals, explains the non-linear and uniform rates of nutrient release [25].

### 3.3 P and K effects on soil

**Fig. 4:**
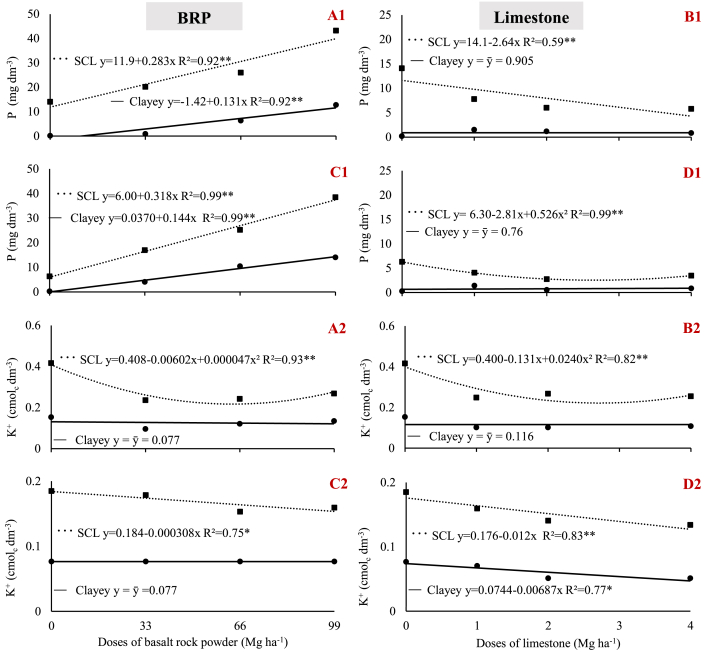
Variation in soil phosphorus (1) and potassium (2) concentrations as a function of BRP and limestone dose. A) BRP with soybean; B) Limestone with soybean; C) BRP with maize; D) Limestone with maize; in sandy clay loam (SCL) and clay (Clayey) soil. ** and * indicate significant differences at 1% and 5% probability, respectively.

Initial soil P concentrations were considered intermediate in the sandy-clay loam and very low in the clay soil (Table 3) [22]. BRP increased soil P to a very high concentration in the sandy-clay loam and to a high concentration in clay soil, regardless of plant species (Fig. 4 A1, and C1) [22]. In contrast, P either remained at very low concentrations (clay) or significantly decreased (sandy-clay loam) upon limestone application, regardless of plant species (Fig. 3 B1, and D1). The variation in soil P may, at least partly, account for the results observed for DM given the increase in DM when using BRP (Fig. 2 A1, and C1) and the weak effect on DM when limestone was applied (Fig. 2 B1, and D1).

The literature shows that soil P availability may increase with increasing pH when limestone is applied [26]. The lack of positive changes in soil P concentration when applying limestone demonstrates that this did not occur under the soil conditions of this study. The soils used in the tests reported herein are not cultivated soils; thus, the amounts of phosphorus fixed in these soils tend to be smaller than that in agricultural soils, and this fixed P fraction corresponds to the P that can be readily released upon pH changes in the soil system.

The effect of BRP on soil P concentration may be associated with two factors other than pH: i) direct P availability; despite the low P concentration in the BRP (Table 1) derived from apatites (0.52%), which are mineralogical constituents of BRP, when applied at high doses, this material provides a significant contribution to soil P content; ii) the release of silicon oxide increase the soil P availability, mainly by competing for phosphate binding sites; so even low P doses applied by rock dusts can cause larger effects on available P [24,[27], [28], [29], [30]].

The increase in soil P concentration resulting from the application of igneous rock powder has been previously reported when assessed using both Mehlich I [5,9,11,15] and resin [13,25].

Initial soil K concentrations were considered ‘high’ in the sandy-clay loam and ‘intermediate’ in clay soil (Table 3) [22]. In the clay soil, none of the materials applied increased the concentrations of this nutrient; on the contrary, K content decreased in the clay cultivated with maize when using limestone (Fig. 4 D2). Similarly, BRP did not increase soil K concentration in any of the two plant species tested (Fig. 4 A2, and C2).

Although no significant increase in soil K is observed in some studies when applying a BRP [4,9,24], other studies have demonstrated that K availability increases when applying a rock powders derived from mafic/ultramafic silicate rocks, in relatively short periods of soil-rock powders interaction, ranging from 45 to 100 days [11,12].

Silva et al. [12] reported that K was released by feldspar found in mafic/ultramafic rocks. In this study, these rocks primarily consisted of microcline minerals (6.12%), containing 3.32% K_2_O, which is considered a high concentration even for rock powders (Table 1). However, this K is not readily exchangeable due to its strong bonds with SiO_4_^−^ and AlO_4_^−^ tetrahedrons, compensating for the deficient charges of these minerals. Thus, K may not be quickly released, and its increase in soil also depends on plant uptake rates. Furthermore, the lower pH of the clay soil may have slightly favored K release from microcline, thus explaining this result.

### 3.4 P and K effects on plants

**Fig. 5:**
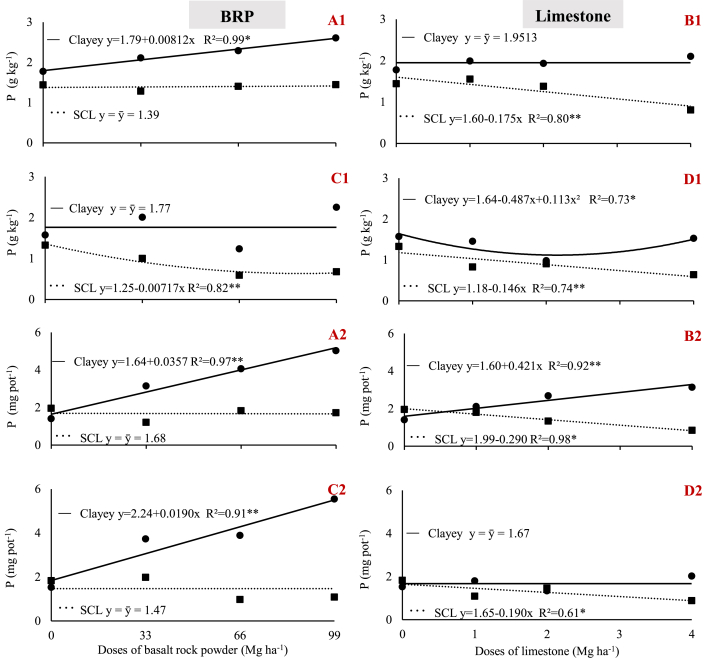
Variation in phosphorus concentration (1) and uptake (2) by plants as a function of BRP and limestone dose. A) BRP with soybean; B) Limestone with soybean; C) BRP with maize; D) Limestone with maize, in sandy clay loam (SCL) and clay soils. ** and * indicate significant differences at 1% and 5% probability, respectively.

Phosphorus availability had significant effects on both crop plant species grown in the clay soil. Thus, soybean showed a significant increase in leaf P concentration, enabling the plant to reach an adequate content (Fig. 5 A1). In turn, maize plants showed concentrations that remained unchanged (1.77 g kg^−1^), and which were deemed adequate (Fig. 5C1). Furthermore, maize leaf P concentrations were similar to those recommended in the literature (1.9 g kg^−1^), albeit whole-plant, not leaf P concentrations.

In the case of the clay soil, P concentration in soybean, but not in maize plants, increased when BRP was applied (Fig. 5 A1, and C1). P uptake increased in both crop species (Fig. 5 A2, and C2). These results, combined with the increase in soil P content with BRP (Fig. 4 A1, and C1), highlight the potential of P availability from this material.

Unlike the clay soil, in the sandy-clay loam, BRP did not increase P concentration in plant tissues, and was below the appropriate range for both crop plants [22]. Maize P concentrations decreased (Fig. 5C1) while P uptake remained unchanged regardless of dose (Fig. 5C2), thus indicating a dilution effect, because maize grown in the sandy-clay loam tended to show an increase in growth (Fig. 2C1).

A hypothesis for these discrepancies between soils may be related to differences in the time of action of the mechanisms of P release from BRP discussed above. Clayey soils, characterized as oxidic, have a high amount of silicon oxides resulting from the high concentration of silicate minerals present in BRP. These silicon oxides, which account for at least 76.42% silicate minerals, affect P release processes more strongly at an early stage, thus actively promoting the release of large quantities of P adsorbed to these minerals, and favoring plant uptake. In turn, BRP apatite can be a source of P for the soil, albeit not fast enough for plant uptake, thus explaining the increase in P in the sandy-clay loam that did not contribute to significant gains of this nutrient by plants.

Limestone had a weak effect on P release in the sandy-clay loam, in which case plant P concentration decreased across treatments and in both crop species (Fig. 5 B1, and D1), thereby decreasing P uptake (Fig. 5 B2, and D2), which in turn explains the lack of gain in DM (Fig. 2 B1, and D1). P uptake by soybean increased in clay soil (Fig. 5 B2) only due to the increase in dry matter (Fig. 2 B1) because leaf P concentration remained unchanged (Fig. 5 B1).

**Fig. 6:**
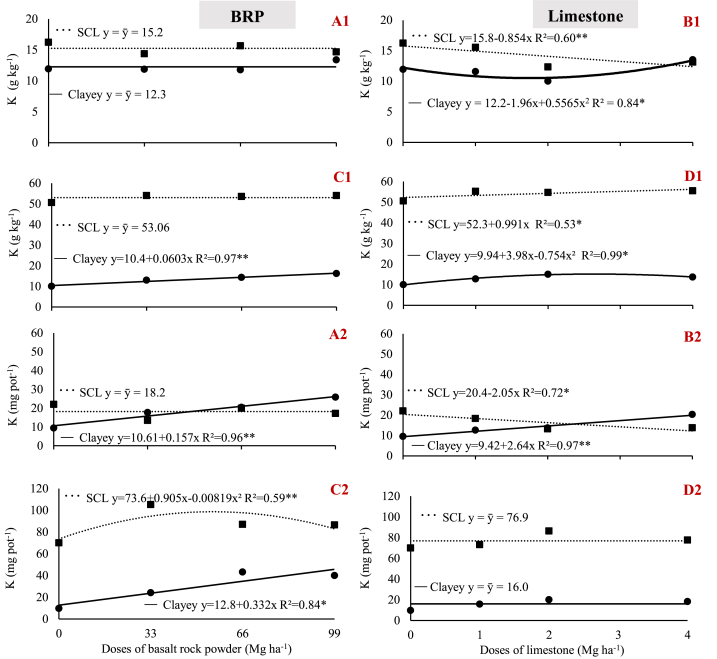
Variation in potassium concentration (1) an uptake (2) by plants as a function of BRP and limestone doses. A) BRP with soybean; B) Limestone with soybean; C) BRP with maize; D) Limestone with maize; in sandy clay loam (SCL) and clayey soils. ** and * indicate significant differences at 1% and 5% probability, respectively.

Meanwhile, in the clay soil, limestone increased the K concentration in maize (Fig. 6 D1), which peaked at 15.2 mg kg^−1^. This value can be deemed satisfactory, as leaf values higher than 17 mg kg^−1^ are considered adequate [22]. The increase in plant K concentration failed to trigger a significant response on K uptake (Fig. 6 D2) but reduced soil K concentration (Fig. 4 D2).

When soils were treated with BRP, this material released enough K to maintain soil K concentrations even when K uptake increased (Fig. 6 A2, C2), as evidenced by the increase in DM production promoted by BRP application (Fig. 2 A1, and C1). The increase in uptake was also accompanied by the increase in the K concentration in maize plants, which reached 16.23 mg kg^−1^ at 99 Mg ha^−1^ (Fig. 6C1). This concentration is only slightly below the value recommended as adequate (17 mg kg^−1^) but was determined in leaves and not in whole plants, as in the literature. In the clay soil, K release from BRP was limited. Nevertheless, this K was assimilated by the crop plants and contributed to the observed increase in DM.

K uptake was higher in maize (82.06 mg pot^−1^) than in soybean plants (17.52 mg pot^−1^), explaining the concentrations found in maize, starting at 0.18 versus 0.42 cmol_c_ dm^−3^ in soil cultivated with soybean (Fig. 4 A2, B2, C2, and D2). Thus, the sandy-clay loam cultivated with soybean managed to maintain its K concentration within the range considered as ‘high’, whereas K concentrations decreased to a level considered as intermediate in the soil cultivated with maize [22].

This difference in uptake is partly explained by the fact that soybean (Fig. 6 A1, and B1) did not reach leaf K concentrations classified as adequate for the crop, ranging from 22 to 27 g kg^−1^, whereas maize K concentrations (Fig. 6C1, and D1) were lower than the recommended range from 17 to 35 g kg^−1^ [22].

K release from BRP differed between soils. In the sandy-clay loam, K release was very low because the variations in K concentration mimicked those observed in soils treated with limestone. In contrast, in the clay soil, K availability was higher when applying BRP.

## 4 Conclusions

BRP significantly increased DM as a function of dose in all treatments, except for soybean sown in the sandy-clay loam. Limestone significantly increased DM of both crop plants only in clay soil, which had a more acidic pH.

BRP and limestone significantly increased soil pH in soybean and maize, but limestone was more efficient because this amendment requires lower doses to achieve the same results.

In terms of bivalent cationic macronutrients, both limestone and BRP significantly increased Ca concentration in both types of soil and in both crop plan species. However, the lower Mg concentration of calcitic limestone and the slower availability of this nutrient in BRP (which is only present in the form of silicate minerals) clearly led to less expressive results, with significant increases of this nutrient only in clay soil treated with BRP and cultivated with soybean.

Phosphorus was significantly increased in soils treated with BRP. This result cannot be due to soil pH increase, since the limestone also increased the pH but had no effect on soil P. Also, the amount of P applied did not explain the result, because the P concentration in BRP was low. The P increase is probably related to interaction of Fe and Al oxides with P and Si, but this hypothesis needs to be further studied, due to the fact that the soil with higher oxides have presented significant increase in extracted P.

Potassium was released from BRP but only under more acidic soil conditions and in amounts insufficient to increase soil K concentration over short periods for its solubilization.

Considering its positive effects on both soil and plants, observed in this study, we conclude that BRP can be an excellent alternative, sustainable, and low-cost nutrient source and growth media for tropical agriculture.

The large number of treatments arising from the variation of soils and crops favors conducting the study in a greenhouse, however this hinders the determination of data such as grain yield, besides long-term soil evaluations, limiting the study to short-term changes. In this way, we highlight the importance of conducting long-term field experiments with rock powders.

The P dynamics in soils treated with rock powders should be better evaluated in trials organized specifically for this purpose. Also, in our research the presence of toxic elements to humans, as well as beneficial trace elements, was not considered, which is an interesting subject that needs to be addressed in further studies.

## Author contribution statement

Robson Fernando Missio: Analyzed and interpreted the data; Wrote the paper.Augusto Vaghetti Luchese: Conceived and designed the experiments; Performed the experiments; Contributed reagents, materials, analysis tools or data; Wrote the paper.Ivone Janete Gutz de Castro Leite; Mylena Linhares Alves; Ana Paula da Silva Giaretta: Performed the experiments.Laércio Augusto Pivetta: Analyzed and interpreted the data; Contributed reagents, materials, analysis tools or data; Wrote the paper.

## Funding statement

This research did not receive any specific grant from funding agencies in the public, commercial, or not-for-profit sectors.

## Data availability statement

Data will be made available on request.

## Declaration of competing interest

The authors declare that they have no known competing financial interests or personal relationships that could have appeared to influence the work reported in this paper.
